# Using 3D Printing Technology to Manufacture Personalized Bone Cement Placeholder Mold for Bone Defect Repair and Reconstruction with Infection: A Case Report

**DOI:** 10.1111/os.13779

**Published:** 2023-06-29

**Authors:** Kai Zheng, Xiu‐chun Yu, Ming Xu, Haocheng Cui, Junyi Wu, Zhiwei Hou, Dongmu Tian

**Affiliations:** ^1^ Department of Orthopedics The 960th Hospital of the PLA Joint Logistice Support Force Jinan China; ^2^ ShanDong Weigao Haixing Medical Device Co., LTD ShanDong China

**Keywords:** 3D Printing, Bone Cement Spacer, Bone Defect, Infection

## Abstract

**Background:**

Limb salvage surgery is the preferred treatment for most malignant bone tumors, but postoperative infection treatment is very challenging. Simultaneously controlling infection and solving bone defects are clinical treatment challenges.

**Case Presentation:**

Here we describe a new technique for treating bone defect infection after bone tumor surgery. An 8‐year‐old patient suffered an incision infection after osteosarcoma resection and bone defect reconstruction. In response, we designed her a personalized, anatomically matched, antibiotic‐loaded, bone cement spacer mold using 3D printing technology. The patient's infection was cured, and limb salvage was successful. In follow‐up, the patient had returned to normal postoperative chemotherapy and was able to walk with the help of a cane. There was no obvious pain in the knee joint. At 3 months after operation, the range of motion of the knee joint was 0°–60°.

**Conclusion:**

The 3D printing spacer mold is an effective solution for treating the infection with large bone defect.

## Graphical abstract

Design and Implementation of the 3D customized antibiotic‐loaded bone cement spacer (ALCS). (A) Based on the original CT data, we printed the four main components of the assembled mold with nylon, with the joint end from metal. (B) ALCS made by mold during surgery had good joint surface morphology. (C) ALCS was successfully inserted to obtain good joint matching, and the incision healed well after operation. (D) Postoperative X‐ray showed that ALCS was in a good position.

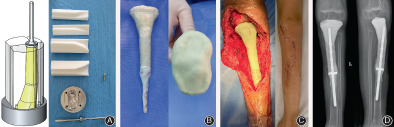

## Introduction

Limb salvage surgery is the standard treatment for most malignant bone tumors around the knee. Reconstructive management for large bone defects and the articular can involve mega‐prosthetic and biological material reconstruction.^[^ ^1^, ^2^, ^3^ ^]^ Whatever the reconstructive management chosen, postoperative infection is a major cause of failure.^[^ ^4^ ^]^ Despite many proposed methods to reduce infection after bone defect reconstruction,^[^ ^5^, ^6^ ^]^ the treatment of bone defect infection (BDI) remains very difficult. Patients often need to undergo multiple surgical treatments, and many patients will even have to undergo amputation due to uncontrolled infection.^[^ ^7^, ^8^, ^9^ ^]^ Some literature suggests that materials for reconstruction of bone defects should be removed during infection treatment.^[^ ^8^, ^9^, ^10^, ^11^ ^]^ Others have proposed a two‐stage revision consisting of complete removal of the mega‐prosthesis or allograft, insertion of the antibiotic‐loaded bone cement spacer (ALCS), and implantation of a new mega‐prosthesis or new components.^[^ ^8^, ^9^, ^11^, ^12^, ^13^ ^]^ ALCS play an important role in the treatment of infection, but traditional ALCS needs to be handmade in surgery. These devices, moreover, have poor appearance and almost no structural function.

Here, we present a new technical method based on 3D printing technology to create custom, anatomically matched ALCS for infection treatment in limb‐salvage applications. There are usually two preparation methods for traditional ALCS. The first method of ALCS is made freehand by the surgeon, and the second is made by a traditional mold. It is difficult to obtain a good appearance for the freehand ALCS, especially for the joint surface. Although traditional molds can compress bone cement to spacers, it is difficult to achieve good personal compatibility. Therefore, we propose 3D printing technology to prepare personalized and fully matched ALCS. In this study, we propose for the first time that the mold adopts a combination structure design and the material selection of nylon for 3D printing, which has short processing time, low processing cost, and convenient clinical application. We present an illustrative case in which we utilized 3D printing technology to save the leg of a child who experienced postoperative infection after osteosarcoma limb salvage treatment. In describing this case, we present the effectiveness of 3D printing ALCS mold abrasives used in surgery and the clinical efficacy of ALCS application.

## Case Presentation

### Clinical Data

#### Diagnosis and Tumor Therapy

**Fig. 1:**
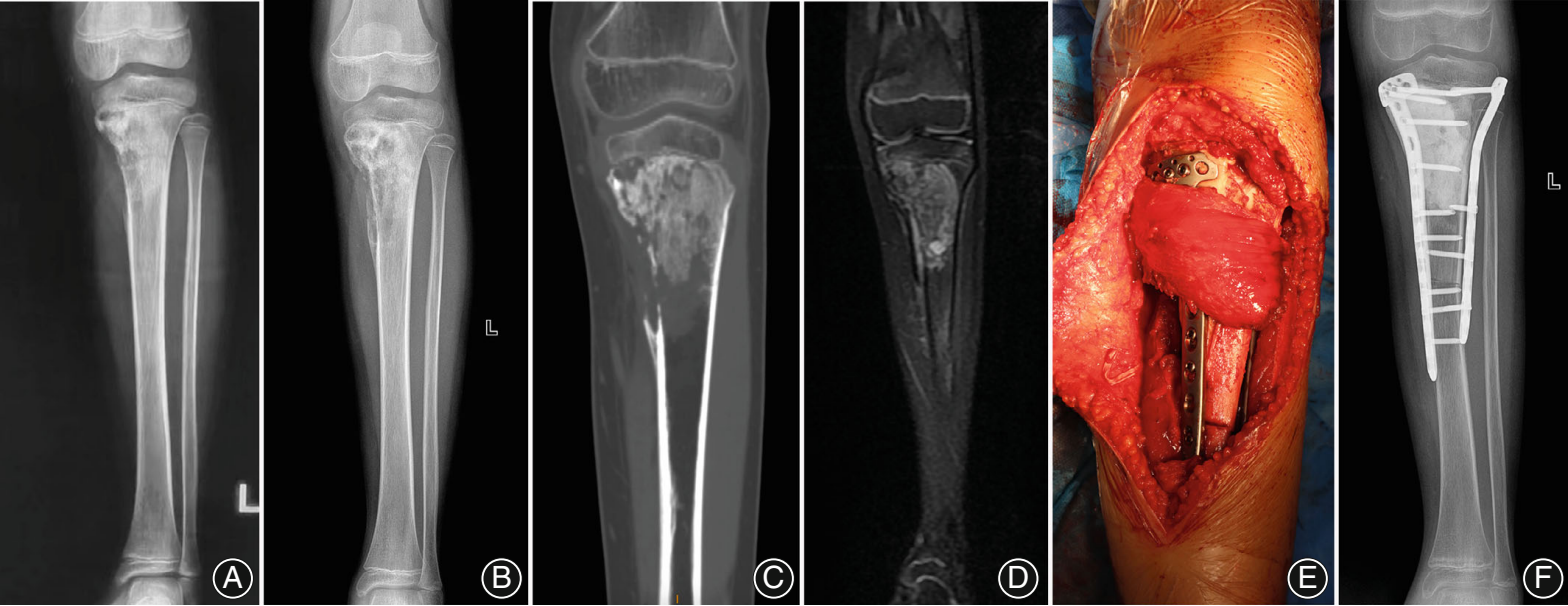
An 8‐year‐old patient with osteosarcoma in the proximal tibia underwent limb salvage surgery. (A): X‐ray at the first visit showed mixed bone destruction in the left proximal tibia. (B): X‐ray showed clear boundary after neoadjuvant chemotherapy. (C and D): After neoadjuvant chemotherapy, CT and MR showed osteogenesis and destruction in tumor mainly and the tumor invaded the epiphyseal plate. (E): After the bone tumor was inactivated and replanted, the gastrocnemius muscle flap was turned over to cover the wound. (F): The inactivated bone and internal fixation were in good position 3 month after operation.

Patient J. K. is an 8‐year‐old girl who suffered from osteosarcoma in the left proximal tibia. After biopsy confirmed her diagnosis, she received two courses of neoadjuvant chemotherapy, including cisplatin, doxorubicin, and ifosfamide. After that, a limb‐salvage operation was undertaken in July 2022 (Figure 1). The first step was to remove the tumor mass with an appropriate margin, along with its surrounding soft tissues. The surrounding bone was then inactivated by microwave ablation, and the necrotic tumor tissue and surrounding soft tissue were completely removed. Thereafter, the weak area of the bone structure was filled and strengthened with bone cement, and the inactivated bone was fixed with bone plate after reimplantation. Postoperative treatment was routine, and the drainage tube was pulled out when the drainage volume was less than 20 mL over 24 h. Postoperative hematological examination showed the following: WBC (white blood cell) 9.35 × 10^9^/L, CRP (C‐reactive protein) 51 mg/L, ESR (erythrocyte sedimentation rate) 20 on the first day. One week later, the values were as follows: WBC 7.98 × 10^9^/L, CRP 8.79 mg/L, ESR 77. Postoperative temperature was normal, and there was no other sign of postoperative infection. Two weeks after the operation, the patient presented with a 2 cm incision in front of her tibia with poor healing, so the sutures in this area were retained while elsewhere they were removed. Postoperative chemotherapy was carried out on schedule, and local small wounds were treated with dressing change.

#### Disease Evolution and Infection

During the second chemotherapy after the operation, the patient's temperature rose abnormally, with a recorded high of 39°. The wound healed poorly with redness and swelling. Secretion from the wound was tested, and bacterial culture tested positive for *staphylococcus aureus*. In response, we halted the patient's chemotherapy regimen, placed her on antibiotic treatment, and performed vacuum sealing drainage treatment on the wound. After 2 weeks of antibiotics, the patient's temperature returned to normal, and the swelling of the nonunion wound had subsided. The patient continued to change the dressing after discharge.

**Fig. 2:**
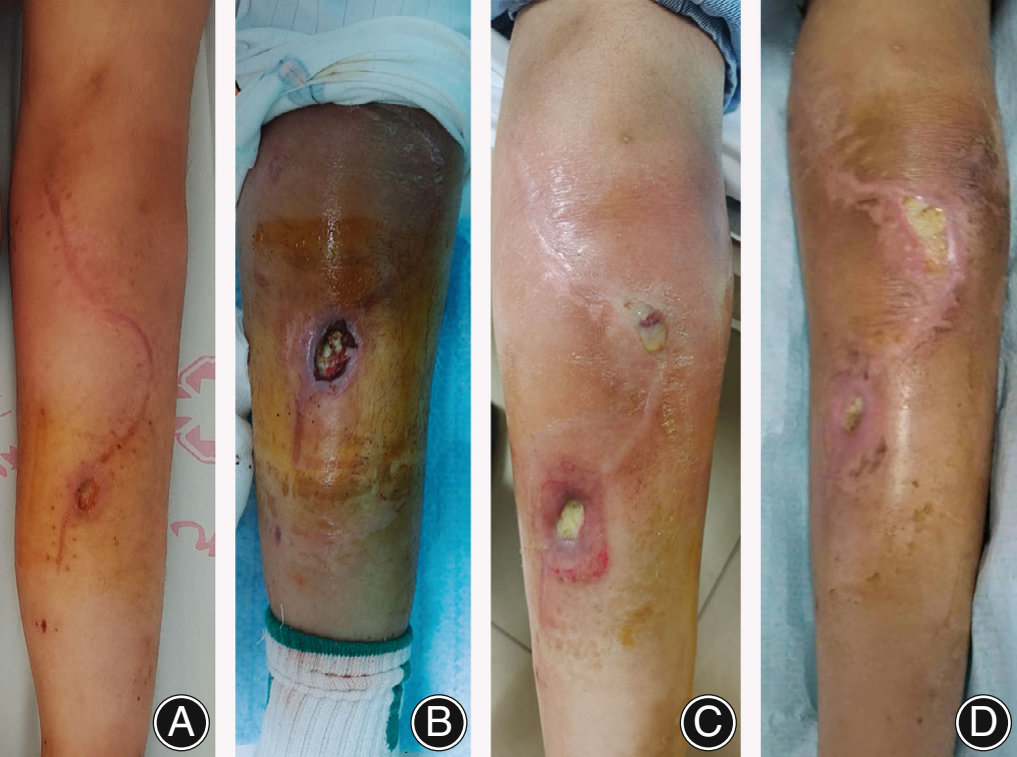
This was the process of infection in the incision. (A): One month after operation, the patient presented with a 2 cm incision in front of her tibia with poor healing. (B): Two months after operation, the wound did not heal, and local infection occurred. (C): Three months after operation, joint infection occurred after the third chemotherapy. (D): After 2 weeks of anti‐infection treatment, the swelling of the knee joint improved, and debridement surgery was planned.

At 3 months after the operation, the patient returned to the hospital for the third round of postoperative chemotherapy. The patient's condition was evaluated as local wound nonunion, and, as no obvious signs of systemic infection were apparent, and the third round of chemotherapy was completed. However, 3 days after the completion of chemotherapy, the patient once again experienced a temperature spike of 39°, local redness and swelling, and knee joint redness and swelling. *Staphylococcus aureus* was again detected by bacterial culture, and WBC 0.87 × 10^9^/L, CRP 143 mg/L, and ESR 109 were detected by blood routine examination. The patient was treated with elevated leukocytes and antibiotics. After 2 weeks of drug treatment, although the patient's white blood cells returned to normal, her temperature still fluctuated between 37° and 38°, her knee joint was still red and swollen, and viscous fluid exuded from the wound, necessitating surgical intervention (Figure 2).

#### Design and Implementation of the 3D Customized ALCS

For this patient, removal of internal fixation and inactivated bone, thorough debridement, placement of ALCS, and secondary joint prosthesis proved effective methods to control infection. Considering that the patient is still in her growth stage, we hoped that the implanted ALCS could also play a certain role in the design of the surgery. Drawing on past clinical experience with children's half joint prosthesis, we used 3D laser printers to make a personalized ALCS mold.

The preoperative design steps are as follows.

**Fig. 3:**
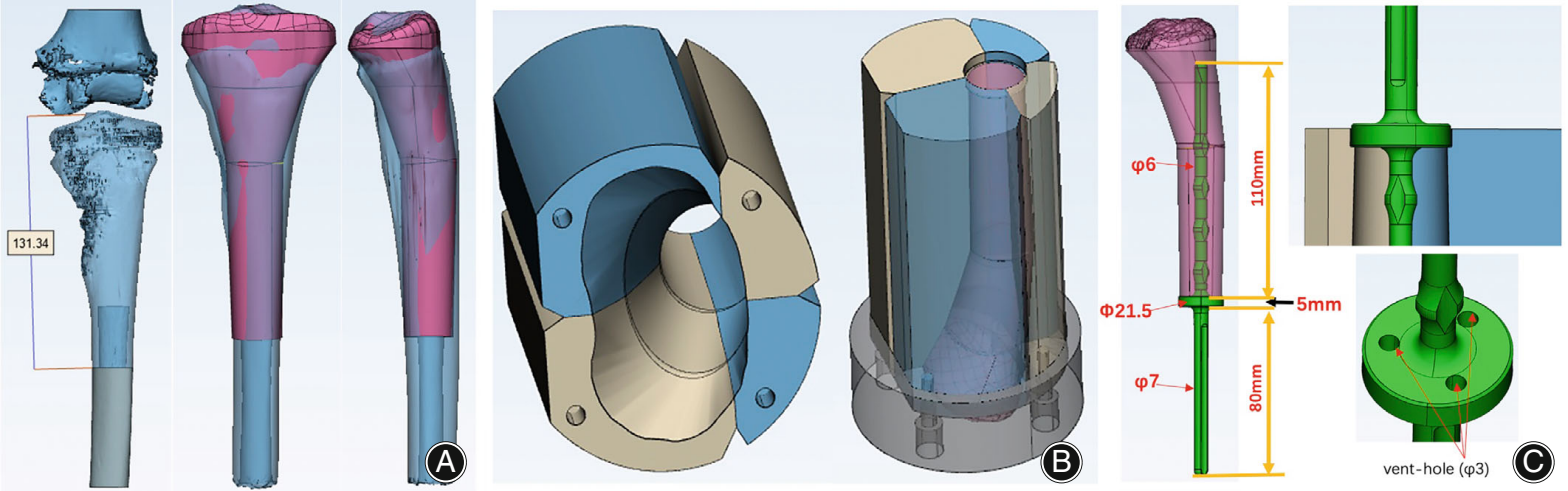
Design and processing flowchart of cement spacer mold. (A): Referring to the initial surgical osteotomy position and the size of the proximal tibia of the healthy limb, optimize the structure and design the size of the spacer. (B): The spacer mold is designed in two parts, with the joint end processed with titanium alloy to obtain a better joint surface, and the other parts printed with nylon material into four components. Nylon components and metal components are fixed with screws to form a complete structure. (C): Design a spacer mold and use it as a medullary needle for internal support and fixation of the cement spacer.

1. Referring to the initial surgical osteotomy position and the size of the proximal tibia of the healthy limb, optimize the structure and design the size of the spacer (Fig. 3A).

2. The spacer mold is designed in two parts, with the joint end processed with titanium alloy to obtain a better joint surface, and the other parts printed with nylon material into four components. Nylon components and metal components are fixed with screws to form a complete structure (Figure 3B).

3. Design a space occupying device and use it as a medullary needle for internal support and fixation of the cement spacer (Figure 3C).

Preoperative experimental use of the model to make a bone cement spacer was successful, and the joint surface shape was satisfactory, faithfully simulating the shape of the autologous tibial joint. To prevent adhesion between the ALCS and the mold, two innovative measures had been applied: first, we applied paraffin oil inside the mold, and second, we took out the ALCS before it was fully solidified.

**Fig. 4:**
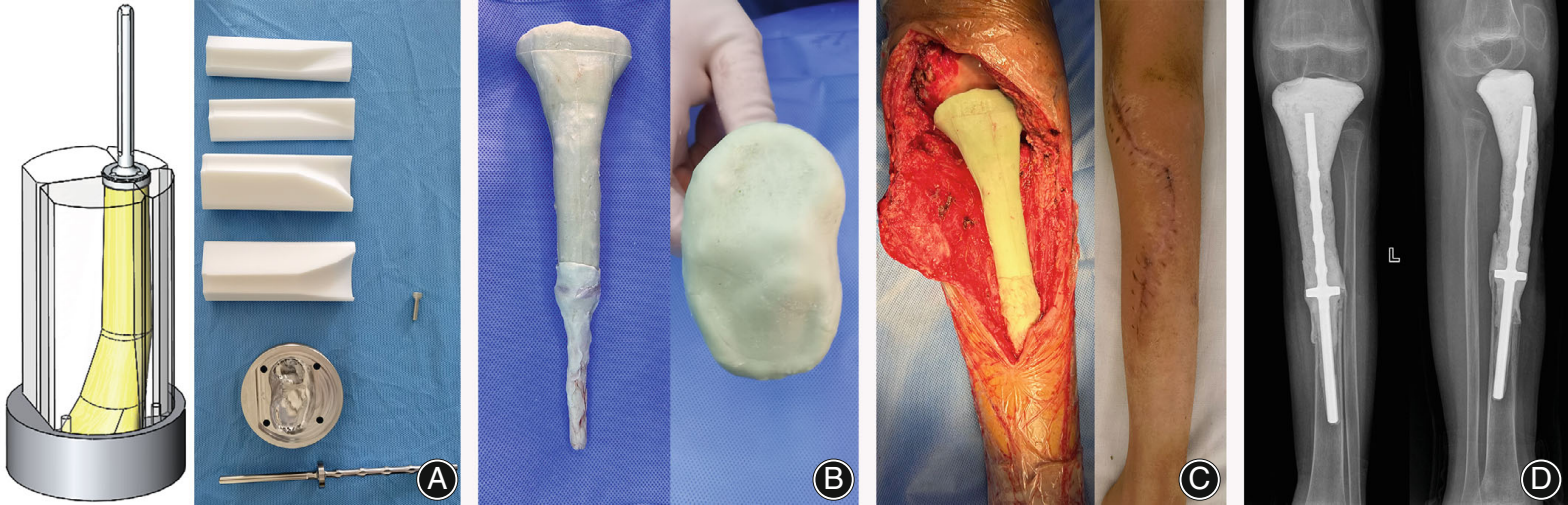
Design and Implementation of the 3D customized antibiotic‐loaded bone cement spacer (ALCS). (A): Based on the original CT data, we printed the four main components of the assembled mold with nylon, with the joint end from metal. (B): ALCS made by mold during surgery had good joint surface morphology. (C): ALCS was successfully inserted to obtain good joint matching, and the incision healed well after operation. (D): Three months postoperative X‐ray showed that ALCS was in a good position.

The patient underwent reoperation according to the preoperative plan in November 2022 (Figure 4). During the operation, a large amount of viscous yellow liquid was found in the joint, which was not connected with the damaged skin. There is also a large amount of inflammatory fluid around the metal plate. There was a slight bony connection between the replanted bone and the host bone. After the screws were removed, the inactivated bone was taken out for thorough debridement. During the operation, a 3D printing mold was used to make an ALCS. After successful production, it was fixed at the site of the bone defect of the proximal tibia in the form of a half joint prosthesis. Following the operation, the wound was rinsed and drained before being reclosed.

#### Postoperative Care

Three days after operation, the patient's temperature returned to normal. The irrigation tube was pulled out 1 week after the operation, and the drainage tube was pulled out 3 days after that. Suture was removed 2 weeks after the operation. At 2, 3, and 4 weeks after the operation, routine blood examination showed that WBC, CRP, and ESR were all normal, so antibiotics were stopped. In total, antibiotics were used intravenously for 32 days. No recurrence of local infection was detected in follow‐up, and the patient returned to normal postoperative chemotherapy. Afterwards, the incision healed normally.

#### Functional Outcomes

The knee joint was fixed with a hinge brace for 6 weeks after operation to promote scar healing of the surrounding soft tissue. The patient could walk with the help of a cane at 3 weeks after operation. Although the patient had lateral instability of the knee joint, it did not affect the patient's walking. The patient was required to wear hinged braces to improve her knee stability. There was no obvious pain in the knee joint. At 3 months after the operation, the range of motion of the knee joint was 0°–60°.

## Discussion

### Infection Control and Bone Defect Repair

In this study, we describe the use of a new spacer for a large bone defect infection revision using 3D printing technology. The 3D printed spacer serves to reduce infection, fill space in the healing bone, and serve as a half joint prosthesis, representing the first use of a device with this sort of multifunctionality. Our clinical results show that this new device reduces the technical complexity of BDI surgery and improves surgical outcomes.

Some studies have explored the clinical effect of ALCS for bone infection, especially for periprosthetic joint infection. For BDI treatment, two‐stage revisions with complete removal of the mega‐prosthesis, ALCS application, and mega‐prosthesis re‐reconstruction showed the best results among limb salvage procedures for the treatment of infected mega‐prosthetics.^[^ ^8^, ^14^ ^]^ The cement spacer mainly fulfills the roles of anti‐infection and space occupation. After infection control, patients need to undergo ALCS removal and new prosthesis implantation. This burdens patients with the ordeal of having to undergo the surgical trauma and greater psychological pressure of receiving two operations within a short time of one another. In addition, the re‐implantation of the prosthesis also brings about the possibility of re‐infection, with a reported re‐infection rate reported in the literature as 22%.^[^ ^11^ ^]^ In this study, we used an anatomically matched, 3D printed ALCS for infection treatment. This new ALCS can perform the function of half joint prosthesis due to its more closely matched shape and superior structural strength. This method extends the use time of the ALCS and avoids the occurrence of reinfection as much as possible. For children still in their growth phase, it is preferable to use the prosthesis for as long as possible, then consider replacing the prosthesis after the patient stops growing.

### 3D Printing Technology on ALCS

At present, 3D printing technology is being increasingly used in orthopaedic medicine. The combination of 3D printing and bone cement space‐occupying devices has been gradually emerging in clinical case reports (Table 1). Tsai et al.^[^ ^15^ ^]^ reported that the use of a computer‐aided design articulating spacer in a two‐staged revision of a periprosthetic knee infection significantly controlled infection, improved clinical outcomes, increased range of motion, and decreased mechanical complications. Kong et al.^[^ ^16^ ^]^ explored a set of alternative articular spacers using 3D printing, which they used in two‐stage revision surgeries for periprosthetic joint infection after total knee arthroplasty. They concluded that 3D printing‐assisted articular spacers provide satisfactory range of motion during the interim period, prevent bone loss, facilitate second‐stage reimplantation and postoperative rehabilitation, and result in low reinfection and complication rates. Broughton et al.^[^ ^17^ ^]^ presented two illustrative cases demonstrating utilization of 3D generated prostheses for limb salvage in the treatment of end‐stage talar pathology complications due to infection. They concluded that this technology has improved limb salvage surgery. Teschke et al.^[^ ^18^ ^]^ reported that a 3D modeled ALCS played an important role in the treatment of mandibular osteomyelitis. Exner et al.^[^ ^10^ ^]^ reported a patient with sarcoma in their forearm who required an uncontaminated resection and control of their infection. A 3D modeled ALCS as an endoprosthetic replacement used in combination with soft tissue reconstruction and systemic antibiotics. They concluded that this novel technique allowed for fast local recovery of the patient's hand function and return to work. In selected cases, such an anatomically formed spacer may be preferred for faster functional recovery, and longer intervals before definitive reconstruction is possible. In this study, the ALCS was applied to the treatment of a proximal tibial bone defect and infection. As far as we know, this is the first time 3D printed spacer molds have been applied to the leg. At this stage, follow‐up results show that the patient is satisfied with the treatment of infection, the limb function has partially recovered, and the patient lacks any overt symptoms of pain.

**TABLE 1:** Clinical report of using 3D printing technology to improve bone cement space‐occupying device.

Author	Year	Disease	Number of cases	3D printing used	Outcome
Kong et al.	2021	Infection after total knee arthroplasty	20	Spacer	Satisfactory function, temporary use
Broughton et al.	2021	Infection of talar fixation	2	Spacer mold	Satisfactory function, mild pain, long‐term use
Exner et al.	2021	Infection of tumor resection in radius	1	Spacer mold	Functional recovery, longer intervals, temporary use
Teschke et al.	2021	Osteomyelitis of the jaw	2	Spacer mold	Satisfactory function, temporary use

### Limitations of this Technique

The approach is, of course, not without risk. The use of ALCS is currently a hot issue. In order to solve this problem, we not only used bone cement in the design, but also titanium alloy support materials. When making the ALCS, we wrapped titanium alloy with bone cement, which increases the strength. The previous ALCS was only used in vivo for a short time, but the safety of long‐term use of ALCS in vivo presents a potential cause for concern. Although the long‐term in vivo use of ALCS lacks safety data, bone cement filling has a long history of application in bone tumor repair and reconstruction. The clinical results show that the long‐term in vivo use of bone cement ordinarily does not cause safety problems.

The long‐term wearing down of the PMMA material's friction interface may loosen the articular spacer and cause instability of the joints. It is difficult to avoid joint surface wear caused by long‐term in vivo ALCS use. Therefore, the ALCS will eventually need be replaced by a mega‐prosthesis. Before performing mega‐prosthesis replacement, it is necessary to consider solving the problem of unequal length of the patient's limbs. If this can be postponed until an individual has reached adolescence and has completed the normal growth and development of his or her limbs, the ALCS will have achieved its purpose. In addition, there are pragmatic considerations to custom printing, mainly those relating to cost and time sensitivity. Production of custom 3D implants and molds may incur additional cost as compared to more traditional treatment options. Given, however, the difficulty of treating postoperative infections in osteosarcoma patients and the severe risk these patients face of needing to undergo an amputation, any kind of limb salvage surgery that can control infection is, in our opinion, worth pursuing. It is better, in our estimation, that treatment only figuratively, rather than literally, costs our patients “an arm and a leg”.

### Conclusion

3D printed ALCS molds have the potential to be a useful tool for temporary or long‐term solutions in the infection with large bone defects. For children and adolescents, this technique may allow for limb‐salvage or temporization until growth arrest and other conditions may be optimized to allow for other reconstructive procedures. Further studies are required to fully establish the clinical efficacy of this scheme.

## Ethics Approval and Consent to Participate

No.

## Authors' Contributions

KZ collected and analyzed the data, prepared the manuscript. JW and HC participated in the surgical operation, collected, and analyzed the data, and prepared the manuscript. ZH prepared the manuscript. MX collected and analyzed the data, prepared the manuscript. KZ and DT designed the mold structure. XY was the principal surgeon, collected and analyzed the data, supervised the writing of the manuscript. All authors have read and approved the manuscript.

## Conflicts of Interest

The authors declare that they have no conflicts of interest pertaining to this study.

## Funding Information

No.
